# Comparison of Posterior and Antero-Lateral Renal Tumors in Retroperitoneal Laparoscopic Partial Nephrectomy: A Propensity Score Matching Analysis

**DOI:** 10.15586/jkcvhl.v10i3.273

**Published:** 2023-07-10

**Authors:** Hakan Anıl, Ali Yıldız, Ahmet Güzel, Serkan Akdemir, Kaan Karamık, Murat Arslan

**Affiliations:** 1 Department of Urology, Adana Seyhan State Hospital, Adana, Turkey;; 2 Department of Urology, Faculty of Medicine, Okan University Hospital, Istanbul, Turkey;; 3 Department of Urology, Aydın State Hospital, Aydın, Turkey;; 4 Department of Urology, Private Tınaztepe Hospital, İzmir, Turkey;; 5 Department of Urology, Korkuteli State Hospital, Antalya, Turkey

**Keywords:** anterior, laparoscopy, partial nephrectomy, posterior, renal masses

## Abstract

This study aimed to compare the antero-lateral and posterior localized renal masses in laparoscopic partial nephrectomy with the retroperitoneal approach in terms of operative, functional, and oncological outcomes. Patients who underwent retroperitoneal laparoscopic partial nephrectomy by a single surgeon between January 2013 and January 2021 were included in the study. A one-to-one propensity score matching (PSM) analysis was conducted to obtain two balanced groups. The patients were divided into two groups as posterior and antero-lateral according to the localization of the mass. A total of 239 patients were included in the PSM analysis, with 65 patients allocated to each group. The mean operative time was 79.2 ± 11.2 min in the posterior group, while it was 90.0 ± 11.6 min in the antero-lateral group (P < 0.001). Warm ischemia time was 15.9 ± 2.4 min in the posterior group and 18.6 ± 2.7 min in the antero-lateral group (P < 0.001). The median decrease in eGFR at 1 year was 4.8 (IQR, 2.9–6.9) mL/min in the posterior group and 5.0 (IQR, 2.8–11) mL/min in the antero-lateral group (P = 0.219). The warm ischemia time and clamping technique were found to be significant factors for predicting eGFR change after surgery (β:0.693, 95% CI: 0.39–0.99, P < 0.001; β:6.43, 95% CI: 1.1–11.7, P = 0.017, respectively). We report that retroperitoneal laparoscopic partial nephrectomy provided longer warm -ischemia and operative time for antero-lateral renal masses than posterior masses. However, long-term oncological and functional results were similar for both localizations.

## Introduction

Partial nephrectomy (PN) is strongly recommended as a standard approach in patients with cT1 renal tumors as it has better functional outcomes and similar oncologic outcomes when technically suitable compared to radical nephrectomy (RN) (1). In the last few years, both robotic and laparoscopic partial nephrectomy (LPN) have emerged as powerful alternatives to open partial nephrectomy due to the demonstration of many advantages such as less blood loss, faster recovery, and fewer complications (2). Also, in recent years, the indications for LPN have gradually expanded to large-scale and stage tumors (pT1b) (1).

Depending on the surgeon’s experience and tumor location, either transperitoneal (TP) or retroperitoneal (RP) approach is preferred (3–5). The benefits of RP–LPN are that it reduces the risk of injury to the intraperitoneal organs and peritoneal irritation caused by postoperative urinary leakage (3, 4, 6). On the other hand, the relative unfamiliarity of the RP approach and limitation of movement of laparoscopic instruments in the small retroperitoneal space may be more technically challenging, especially for anterior renal masses. Moreover, the retroperitoneal approach to tumors located antero-medially is much more of a challenge and requires excessive renal mobilization. It was reported that patients with antero-medial tumors are not suitable candidates for the RP approach (7). In the context of laparoscopic surgery, the difficulties and efficacy of the RP approach for antero-lateral renal masses compared to posterior renal masses have not been fully studied.

Therefore, in our study, we aimed to compare the perioperative, functional, and oncological results for patients with posterior and antero-lateral renal masses treated with LPN by a single surgeon experienced in the RP approach.

## Materials and Methods

### Study population

Following institutional ethical committee approval, clinical data collected for 259 consecutive patients with localized renal masses who underwent retroperitoneal LPN by a single surgeon (M.A.) in two centers between January 2013 and January 2021 were retrospectively evaluated. Patients with congenital renal malformation (n = 1), solitary kidney (n = 2), coagulation disorders (n = 9), and radiofrequency ablation history (n = 1), and those with missing data (n = 7) were excluded. A total of 239 patients were included in the analysis.

The demographic and clinical characteristics of patients including data such as age, gender, body mass index (BMI), American Society of Anesthesiologists (ASA) score, comorbidities (such as diabetes and hypertension), smoking status, estimated glomerular filtration rate (eGFR), and tumor size were recorded. Preoperative creatinine levels were measured routinely 3–10 days before the surgery.

Preoperatively, abdominal CT scan and/or MRI were taken for all patients in order to assess tumor characteristics and clinical TNM staging. The R.E.N.A.L. (radius; exophytic/endophytic; nearness; anterior/posterior; location) nephrometry scoring system was used to assess the tumor complexity for each renal mass. The anterior and posterior distinction of tumor localization was made according to the R.E.N.A.L. nephrometry scoring previously described in the literature (8). Another line was drawn steeply intersecting the anterior or posterior line. Thus, the kidney was divided into four quadrants: posterolateral, posteromedial, anterolateral, and anteromedial. If the tumor was in two quadrants, it was included in the quadrant group with more than 50% tumor burden. The R.E.N.A.L. nephrometry scores and tumor localization were calculated retrospectively by a clinician who was blinded to the patient characteristics and surgical outcomes.

### Surgical technique

As with previous descriptions of the RP–LPN technique, under general anesthesia and in the full flank position, the RP cavity was bluntly entered using the index finger with a 16–18 mm transverse incision under the tip of the 12th rib in Petit’s triangle, then the cavity was expanded using an optical dilatation balloon (9). A 12 mm trocar for the right hand and a 5 mm trocar for the left hand were inserted into the RP cavity. Gerota’s fascia was incised horizontally parallel to the psoas muscle to dissect the renal artery by reaching the renal hilum. To localize the renal mass, the entire kidney was isolated from perirenal fat tissue (excluding the fat overlying the tumor). If more than 50% of the tumor was exophytic and smaller than 3 cm, the decision was made to perform the off--clamping technique during preoperative evaluation. However, the final decision was made according to intraoperative findings. The tumor was identified macroscopically and pulled out by a cold scissors with 4–5 mm of the parenchymal margin. The tumor specimen was enclosed in an endoscopic bag. The tumor bed was closed with a running 3-0 V-Lock suture, and renal parenchyma was closed with an interrupted 2.0 polyglactin suture. Finally, the surgical specimen bag was removed from the optic trocar incision and roughly examined, a drain (24 Fr) was inserted through the 12 mm port incision, and the port incisions were closed by suturing. The surgeries were performed by a single surgeon (MA) working at a tertiary university hospital and experienced in urological laparoscopy.

### Postoperative assessment

Perioperative and postoperative data of the patients, including operative time (OT), warm ischemia time (WIT), open surgery conversion rate, estimated blood loss (EBL), pre- and postoperative hematocrit values, length of hospital stay (LOS), final pathology, pathological stage, surgical margin involvement, and complications were recorded and compared according to tumor location. The classification of recorded complications was made according to the Clavien-Dindo system (10). eGFR was calculated using the Chronic Kidney Disease Epidemiology Collaboration (CKD-EPI) equation using preoperative and postoperative first-year serum creatinine levels. Renal functional outcomes were represented by the absolute change in eGFR. The absolute change in eGFR was defined as preoperative eGFR - postoperative eGFR at 12 months.

Finally, the validated M.I.C. system (negative surgical margins, WIT <20 min, and no major complications [Clavien III/IV]) was used to evaluate the success rate of partial nephrectomy. In addition to this trifecta system, the condition of the patient was evaluated 1 year later using the pentafecta system, which is defined as >90% preservation of eGFR and no upgrading of chronic kidney disease until 12 months after LPN.

### Statistical analysis

Data are expressed as n (%), mean ± standard deviation, or median and interquartile range (25th–75th, IQR), as appropriate. The normality assumption was checked with the Kolmogorov–Smirnov test. The Mann–Whitney U test or Student’s t-test was used for continuous variables. Pearson chi-square or Fisher’s exact test was performed for categorical variables. Multivariate linear regression analysis was performed to identify predictive factors for GFR changes after surgery. Potential imbalances between groups were expected because of the study design. A one-to-one propensity score matching (PSM) analysis was conducted to reduce potential bias and to attain comparable groups. A caliper of 0.20 of the standard deviation of the logit of the propensity score was used to obtain similar groups regarding the set of covariates. Statistical assessments were performed using SPSS software pack (Statistical Package for Social Sciences for Windows version 22 software; IBM Corp., Armonk, NY) and R program (version 2.15.2 for Windows). To incorporate these programs and to perform PSM analysis, a developer-based software providing a custom dialog in the SPSS menu was used (11). P < 0.05 was considered statistically significant.

## Results

**Figure 1:**
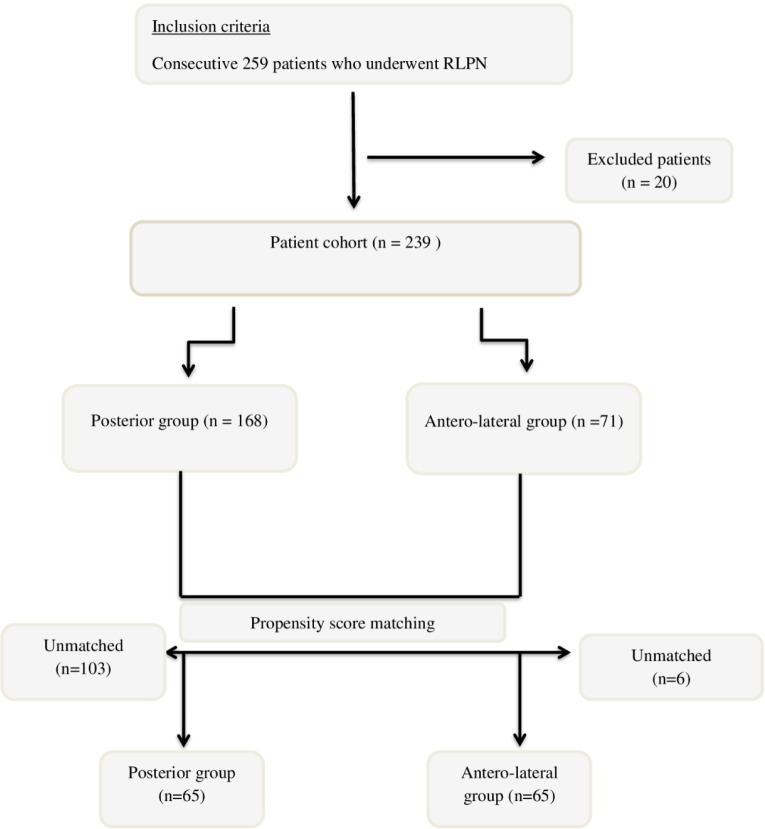
Patient selection flow chart.

A total of 130 patients, 65 with posterior and 65 with antero-lateral renal masses, were included in the analysis after PSM (Figure 1). The distribution of the preoperative outcomes in unmatched and matched cohorts are shown in Table 1.

**Table 1:** The comparison of study groups in terms of pre-operative variables before and after matching.

	Unmatched comparisons		P	Matched comparisons		P
	Posterior (n = 168)	Antero-lateral (n = 71)		Posterior (n = 65)	Antero-lateral (n = 65)	
Age, years, mean ± SD	54.9 ± 10.5	59.8 ± 8.1	0.001	58.5 ± 11.2	59.3 ± 8.2	0.638
Female patients, n (%)	46 (27.4)	20 (28.2)	0.901	22 (33.8)	18 (27.7)	0.447
BMI, kg/m^2^ (mean ± S.D)	29.2 ± 3.6	28.1 ± 3.1	0.022	27.9 ± 3.4	28.2 ± 3.1	0.649
ASA score, (median, IQR)	2 (1.8–2.05)	2 (2–2)	0.684	2 (1.95–2)	2 (2–2)	0.614
Presence of Hypertension, n (%)	47 (28)	22 (31)	0.639	15 (23.1)	21 (32.3)	0.240
Smoking, n (%)	89 (53)	36 (50.8)	0.748	35 (53.8)	31 (47.6)	0.483
GFR value, mean ± SD	85.5 ± 16.4	90 ± 20.1	0.102	89.6 ± 18.7	89.2 ± 20.4	0.912
Preoperative CKD stage, n (%)	–	–	0.001	–	–	0.369
Grade 1	45 (26.8)	32 (45.1)	–	26 (40)	29 (44.6)	–
Grade 2	112 (66.7)	29 (40.8)	–	33 (50.8)	26 (40)	–
Grade 3	11 (6.5)	10 (14.1)	–	6 (9.2)	10 (15.4)	–
R.E.N.A.L. nephrometry score, (median, IQR)	5.5 (5–6.4)	5 (4–6)	0.168	5 (4–6)	5 (4–6)	0.769
Tumor size, cm, (mean± SD)	4.2 ± 1.5	3.6 ± 1.5	0.007	3.7 ± 1.6	3.5 ± 1.5	0.601
Clinical T Stage, n (%)	–	–	<0.001	–	–	0.520
cT1a	74 (44)	56 (78.9)	–	46 (70.8)	50 (76.9)	–
cT1b	86 (51.2)	13 (18.3)	–	18 (27.7)	13 (20)	–
cT2a	8 (4.8)	2 (2.8)	–	1 (1.5)	2 (3.1)	–

The mean operative time was 79.2 ± 11.2 min in the posterior group and 90 ± 11.6 min in the antero-lateral group (P < 0.001). Warm ischemia was applied to 41 (63.1%) patients in both groups. The mean duration of warm ischemia was 15.9 ± 2.4 min in the posterior group, while it was 18.6 ± 2.7 min in the antero-lateral group (P < 0.001). In three procedures, which started with off-clamping in the antero-lateral group, warm ischemia was reversed due to intraoperative bleeding. None of the patients in either group converted to open surgery or radical nephrectomy. Clavien-Dindo Grade 1 complications were observed in five patients of the posterior group and three patients of the anterolateral group. For the anterior group, four out of five patients had transient elevation of serum creatinine and one patient had fever. For the antero-lateral group, three patients had transient elevation of serum creatinine. Due to the decrease in hemoglobin in the postoperative period, blood transfusion (Clavien-Dindo Grade 2) was required in one patient in the posterior group and two patients in the antero-lateral group. A double J stent was inserted (Clavien-Dindo Grade 3a) after urine extravasation from the collecting system in one patient in the antero-lateral group. The results for intraoperative, perioperative, postoperative, and early complications are presented in Table 2.

**Table 2:** Comparison of intra-preoperative outcomes according to groups.

	Posterior group (n = 65)	Antero-lateral group (n = 65)	P
***Intraoperative***			
Operative time, min, mean ± S.D	79.2 ± 11.2	90.0 ± 11.6	<0.001^*^
Warm ischemia time, min, mean ± S.D	15.9 ± 2.4	18.6 ± 2.7	<0.001^*^
Ischemia technique, n (%)	–	–	1.000^**^
Warm ischemia	41 (63.1)	41 (63.1)	–
Off clamping	24 (36.9)	24 (36.9)	–
Estimated blood loss, mL, median(IQR)	200 (150–250)	175 (100–300)	0.802^***^
***Peroperative***			
Hospital stay, day, mean ± S.D	3.1 ± 0.5	3.1 ± 0.6	0.885^*^
Drain removal time, day, mean ± S.D	2.06 ± 0.2	2.1 ± 0.5	0.407^*^
Htc drop, median (IQR)	4.6 (2.6–7.9)	4.5 (2.8–9.0)	0.385^***^
***Postoperative***			
***Complications within 30 days, n (%)***			
None	59 (90.8)	59 (90.8)	–
Clavien Dindo Grade 1, n (%)	5 (7.7)	3 (4.6)	–
Clavien Dindo Grade 2, n (%)	1 (1.5)	2 (3.1)	–
Clavien Dindo Grade 3a, n (%)	0 (0)	1 (1.5)	–
Tumor pathologic grade	–	–	0.888^**^
Grade 1, n (%)	43 (70.5)	42 (70)	–
Grade 2, n (%)	15 (24.5)	16 (26.7)	–
Grade 3, n (%)	3 (5.0)	2 (3.3)	–
MIC score, n (%)	61 (93.8)	58 (89.2)	0.344^¥^
Pentafecta, n (%)	48 (73.8)	43 (66.1)	0.338^**^
Follow- up time, months, median (IQR)	51 (42–69)	46 (38–60)	0.015^***^

MIC, margin, ıschemia, complication; SD, standard deviation; IQR, interquartile range; ^*^ denotes Student’s t test, ^**^ denotes chi-sqaure test, and ^***^ denotes Mann–Whitney U test.

When the final pathology results were evaluated, surgical margins were positive in four (4.3%) patients in the posterior group and four (5.6%) patients in the antero-lateral group (P = 0.706). The histological types were clear cell carcinoma for 92 (70.8%), papillary carcinoma for 19 (14.6%), oncocytoma for 8 (6.2%), chromophobe for 5 (3.8%), angiomyolipoma for 3 (2.3%), and cystic-solid for 3 cases (2.3%). Recurrence was detected in one patient in both groups during mean follow-up of 51.1 ± 15.8 months.

The median absolute change in eGFR at 1 year was 4.8 (IQR, 2.9–6.9) mL/min in the posterior group and 5.0 (IQR, 2.8–11) mL/min in the antero-lateral group (P = 0.219). Warm ischemia time and clamping technique were found to be significant factors for predicting eGFR change after surgery (β:0.693, 95% CI: 0.39–0.99, P < 0.001; β:6.43, 95% CI: 1.1–11.7, P = 0.017, respectively ) (Table 3).

**Table 3:** Identifying to predictive factors for GFR changes after surgery with univariate and multivariate linear regression model.

		Univariable			Multivariable	
	β	95% CI	P	β	95% CI	P
Age, years	0.023	-0.08, 0.11	0.791	0.010	-0.071, 0.09	0.806
BMI, kg/m^2^	0.340	-0.23, 0.35	0.700	0.099	-0.14, 0.34	0.416
R.E.N.A.L. nephrometry score	0.169	-0.16, 1.39	0.055	0.238	-0.35, -0.83	0.431
Tumor location						
Posterior	Reference					
Antero-lateral	0.172	0.01, 3.84	0.050	0.482	-1.21, 2.18	0.575
Ischemia technique						
Unclamping	Reference					
Clamping	5.34	3.55, 7.13	0.001<	6.43	1.15, 11.7	0.017
Warm ischemia time, mins	0.36	0.26, 0.45	0.001<	0.693	0.39, 0.99	0.001>

## Discussion

The retroperitoneal approach for laparoscopic partial nephrectomy is chosen less often by urologists, in spite of reporting lower complications, hospital stay, and operative duration (12). Though RP-LPN provides an advantage for posterior masses, it involves anatomic difficulties for anteriorly localized masses. The main finding of this study is that the retroperitoneal approach in laparoscopic partial nephrectomy is an effective and safe approach for renal masses with antero-lateral localization.

Transperitoneal access is widely preferred in patients with anterior renal masses because it allows a large working area (3, 4). However, RP access is an alternative option in patients with previous abdominal surgery and posterior renal mass (3–5, 13). Although narrow working area and limited exposure of medial kidney masses are disadvantages of the retroperitoneal approach, its advantages are that it allows immediate and direct access to renal vessels, posterior hilar structures, and posterior kidney masses without excessive mobilization and rotation of the kidney (3–6, 13). While both approaches were reported to be safely applicable during LPN, efficacy results in studies comparing both approaches are conflicting due to differences in tumor location (3–5). In three studies comparing the two approaches for posterior tumors, it was reported that only the length of hospital stay and operative time were shorter with the RP approach (13–15).

In a study comparing retroperitoneal robot-assisted partial nephrectomy (RP-RAPN) for posterior and -antero-lateral masses, the RP approach was identified to be a safe and effective method for posterior masses in addition to -antero-lateral masses (7). Limitations of the study include short follow-up duration, low number of subjects, and lack of oncologic outcomes. In our study, in addition to reporting that RP-LPN is effective and safe for renal masses with -antero-lateral localization, it was also reported to be effective on long-term oncologic outcomes.

In the study by Harris et al., they compared the transperitoneal approach in RAPN for antero-lateral and posterior masses (16). They reported no significant differences in terms of warm ischemia duration, operative duration, and complications. In our study, while there was no difference in terms of complications between posterior and anterior masses, the warm ischemia and operation durations were in favor of posterior masses. Though this difference was significant, the warm ischemia duration in the anterior group was within acceptable intervals. Tanaka et al. compared transperitoneal RAPN (TP-RAPN) and RP-RAPN cases in a retrospective study that and found surgical duration and predicted bleeding time were in favor of the retroperitoneal approach (17). However, in this study, there were no anterior renal masses in the RP-RAPN group, and the sample number was low.

Another retrospective two-center comparison of TP-RAPN and RP-RAPN reported that surgical durations were statistically significantly in favor of RP-RAPN (18). Pentefacta rates were similar in both groups. In this study, though 16% of patients in the retroperitoneal group had mass with anterior localization, anterior or posterior comparison results were not reported within the group. It was emphasized that anterior and posterior masses may be treated with both methods; here, the key factor is the experience of the surgeon. Hark et al. reported that surgical and warm ischemia durations and complication rates were in favor of RP-RAPN in multicenter matched pair analysis studies (19). Of the 116 cases in the retroperitoneal approach group, only 20 had anterior mass. According to tumor location in the RP-RAPN group, surgical duration and complication rates were lower for posterior masses, while warm ischemia durations were similar.

Variation in GFRs after partial nephrectomy is a basic parameter for assessment of functional outcomes. The targeted functional outcome is eGFR change of less than 10% after partial nephrectomy (20). Factors affecting the variation in eGFR may be listed as long ischemia duration, excess blood loss, low preoperative GFR levels, and high nephrometry score (21). In our study, the most important factor affecting eGFR was identified to be warm ischemia duration. Tumor diameter, location, R.E.N.A.L. nephrometry score, and other factors were not found to be effective risk factors for eGFR.

Our study also had some limitations. The main limitation was its retrospective design, which may have led to the possibility of misclassification bias. The relatively small sample size was also a major limitation. However, in order to compensate for potential cofounders and preoperative imbalances, we performed PSM analysis. Additionally, the follow-up duration was also sufficiently long.

## Conclusion

RP-LPN is known to provide many advantages for posterior renal masses. In this study, we report that although the operative time and warm ischemia time are longer for antero-lateral masses compared to posterior-located masses, RP-LPN is also an effective and safe approach for antero--lateral masses in terms of oncological and functional outcomes.

## Authors Contribution

HA contributed to the project development, data collection, data analysis, and manuscript writing and editing. AG was involved in project development and manuscript writing and editing. AY contributed to the project development and data collection. SA helped with data collection and manuscript writing and editing. KK was involved in data collection and manuscript writing and editing, and MA contributed to the project development and manuscript writing and editing.
